# Effect of warfarin versus aspirin on blood viscosity in cardioembolic stroke with atrial fibrillation: a prospective clinical trial

**DOI:** 10.1186/s12883-019-1315-5

**Published:** 2019-05-01

**Authors:** Chan-Hyuk Lee, Keun-Hwa Jung, Daniel J. Cho, Seul-Ki Jeong

**Affiliations:** 1 Department of Neurology, Seoul National University Hospital, Seoul, South Korea; 2 Department of Neurology, Chonbuk National University Hospital, Jeonju, South Korea; 3 Rheovector, LLC, King of Prussia, PA USA; 4 Department of Neurology & Research Institute of Clinical Medicine, Chonbuk National University Medical School - Biomedical Research Institute of Chonbuk National University Hospital, Geonjiro 20, Deokjin-gu, Jeonju, Chonbuk 54907 South Korea

**Keywords:** Atrial fibrillation, Cardioembolic stroke, Warfarin, Whole blood viscosity

## Abstract

**Background:**

Warfarin is evidence-based therapy for the prevention of cardioembolic stroke, but has not been studied for its effects on whole blood viscosity (WBV). This study investigated the effect of warfarin versus aspirin on WBV in patients presenting with non-valvular atrial fibrillation (NVAF) and acute cardioembolic stroke.

**Methods:**

We enrolled patients with acute cerebral infarction, aged 56–90 years who had NVAF, CHADS_2_ score ≥ 2, presenting with mild-to-moderate stroke (National Institute of Health Stroke Scale (NIHSS) score < 20 and modified Rankin Scale (2mRS) score < 4) in a single center. The patients were alternately assigned to warfarin or aspirin groups. Post-treatment WBV was assessed after international normalized ratio (INR) reached target range [2, 3] for patients in the warfarin group, and 5 days after baseline in the aspirin group.

**Results:**

Total 67 patients were included, and 56 completed this study (33 warfarin and 23 aspirin). Compared to baseline values, warfarin reduced post-treatment BV at all shear rates. The BV reductions greater than 1 cP measured at shear rates of 300, 150, 5, and 1 s^− 1^ were independently and significantly associated with warfarin treatment compared to aspirin after adjusting for age, sex, CHA_2_DS_2_-VASc scores, and baseline hematocrit.

**Conclusions:**

Warfarin confers greater reductions in BV than aspirin in patients with acute cardioembolic stroke. BV could be a useful method to estimate thrombotic risk in patients receiving warfarin.

**Trial registration:**

KCT0001291, Date of Registration: 2014-12-01

## Background

Whole blood viscosity (WBV) is defined as the internal resistance of blood flow and it determines the frictional force applied to the vessel wall. The WBV has been reported to be independently associated with the cardiovascular risk factors, such as hypertension, diabetes, hyperlipidemia, obesity, and cigarette smoking [1]. The previous epidemiologic studies have shown that the WBV was significantly and positively associated with the higher occurrence of major cardiovascular events, including cardiovascular death [2, 3]. The WBV is a component to calculate arterial wall shear stress which is a biomechanical tangential force along the arterial wall [4, 5]. Mathematically, it can be simplified to τ = μ ⨯ γ. Where τ is the shear stress, μ is the viscosity, and γ is the shear rate [5]. Shear rate is the change in blood velocity relative to the inner diameter of the vessel and ranges between 20 and 500/s. Shear stress is the frictional resistance of the blood to the blood vessel walls.

Warfarin is evidence-based therapy for the prevention of cardioembolic ischemic stroke in patients with non-valvular and valvular atrial fibrillation (AF). If concentration of warfarin in blood does not reach therapeutic levels in patients with AF, ischemic stroke cannot be effectively prevented [6] and if warfarin is overdosed, bleeding tendency is increased [7]. Therefore, proper use of warfarin is important to prevent cardioembolic stroke. International normalized ratio (INR) is widely used as a means of monitoring the effect of warfarin. However, the results may differ for each reagent manufacturer used for the INR measurement, as international sensitivity index (ISI) is required to calculate INR, which demonstrates the variability of the INR value even for the same sample [8–10]. Previous studies have also shown that INR variability is also high in the external quality assurance data. In addition, it has been reported that the laboratory INR and the point-of-care INR were different from each other [11, 12].

Considering the pharmaceutical effects of warfarin on blood clotting factors, we assumed that it could affect the levels of the WBV according to the concentration of the drug. No previous study has examined whether the WBV could be changed in the therapeutic ranges of warfarin in patients with atrial fibrillation. Meanwhile, there were studies on the association between aspirin and WBV. There was no statistically significant difference of WBV between the aspirin group and the placebo group in the unadulterated blood sample [13]. Other studies that measured blood viscosity and red cell deformability in healthy adults did not show any difference between before and after aspirin administration [14].

For the purpose, this study investigated the effects of warfarin and aspirin on blood viscosity in cardioembolic ischemic stroke patients with non-valvular atrial fibrillation (NVAF).

## Methods

### Patient population

This is a prospective, alternately assigned, open-label and blinded-endpoint clinical trial. We enrolled patients aged 56–90 years in acute stage of ischemic stroke. Presentation of acute ischemic stroke was defined as high signal intensity in a diffusion weighted image and low signal intensity in an apparent diffusion coefficient simultaneously with brain magnetic resonance image among patients experienced symptoms of stroke within 7 days. Transthoracic echocardiography was performed on patients with AF identified in the electrocardiography, and only patients with non-valvular type were finally enrolled in the study. In addition, according to the Trial of Org 10,172 in Acute Stroke Treatment (TOAST) classification, we further confirmed whether the patient was eligible for cardioembolic stroke.

Among the patients, only individuals with mild-to-moderate stroke (National Institute of Health Stroke Scale (NIHSS) score < 20 and modified Rankin Scale (mRS) score < 4) were selected to exclude patients with severe neurological deterioration. Patients with severe stroke with high NIHSS often have large ischemic stroke [15], and have an increased risk of hemorrhagic transformation. This increases the possibility of alteration or discontinuation of antithrombotic agents [16].

In order to select only patients who need anticoagulation, we reaffirmed whether CHADS_2_ score is over 2. (CHADS_2_ score: C, congestive heart failure; H, hypertension; A, ≥75 years; D, diabetes mellitus; S_2_, prior stroke, transient ischemic attack or thromboembolism).

Patients were excluded if they were pregnant or lactating, previously treated with anticoagulants or steroids, or if they had hemorrhagic stroke, endocarditis, severe mitral stenosis or mechanical heart valve replacement, hematologic, hepatic, renal diseases or malignancy. Additionally, transient atrial fibrillation after surgery, high risk of bleeding, hemoglobin < 7.0 mg/dl, probability of blood transfusion during the trial, platelet > 450,000/μL, platelets < 90,000/uL, uncontrolled diabetes mellitus and hypertension were excluded from the study. Patients were alternately assigned according to the order of enrollment, and the warfarin group was set to open-label study for appropriate INR control. This study was approved by the Institutional Review Board of the Biomedical Research Institute of Chonbuk National University Hospital and registered in Clinical Research Information Service in Korea (CRIS Reg No. KCT0001291). All participants provided written informed consent.

### Laboratory data

Complete blood counts, blood lipid profile, renal function, liver function, HbA1c, urine albumin-to-creatinine ratio, homocysteine, and blood coagulation factors such as prothrombin time (PT), activated partial thromboplastin time (aPTT), and D-dimer were tested using standard methods. In particular, the blood for the INR, PT, and aPTT is contained in a sodium citrate tube, and the plasma is automatically analyzed using an ACL TOP (Werfen, Australia) analyzer.

To measure the WBV, 3–5 mL of whole blood from each patient was collected from peripheral vein and agitated gently in ethylenediaminetetraacetic acid (EDTA) tube. The blood was refrigerated at 4 °C until analysis. The WBV was measured using a computerized scanning capillary viscometer (Hemathix, King of Prussia, PA) which provided the WBV at shear rates of 1 to 1000/s at increments of 0.1/s. The WBV was measured within 36 h of sampling, and the blood was maintained at 37 °C during the analysis.

### Outcome measure and sample volume estimation

The primary outcome measures were changes in WBV at high and low shear rates (300 and 5/s respectively). For the purpose of that, baseline WBV was assessed before treatment. Post-treatment WBV was assessed after reaching the therapeutic range (INR 2–3) for patients in warfarin group. Aspirin group received 300 mg of aspirin on the first day, and 100 mg was given for the next 5 days before blood analysis.

All the patients who were enrolled in the present study began to receive warfarin within 14 days after the onset of neurological symptoms which was in accordance with the current guidelines [17].

In a previous study using scanning capillary viscometer in patients classified as high-risk for cardiovascular disease based on a Framingham risk equivalent of > 20%, standard deviation of mean WBV levels at low shear rate (5/s) were reported as 2.42 cP [18]. We enrolled 50 subjects to detect a change of one standard deviation (SD) in mean low-shear blood viscosity or 25 subjects in each of 2 arms using 90% power and a type 1 error rate of 5%.

### Statistical analysis

Descriptive data for the major characteristics were expressed as means ± SDs or percentages as appropriate. Independent t-test or chi-square test was used to determine the statistical differences between the two groups. For the WBV, paired t-tests (Wilcoxon signed rank tests) were performed to compare the treatment effects. For a logistic regression analysis, blood viscosity reductions 1 cP or more was set to be a dependent variable and the association with treatment modality was examined for an independency. Statistical analysis was performed by the third-party statistician and conducted using SAS (SAS, Cary, NC) and SPSS Statistics 20 (SPSS, Chicago, IL).

## Results

### Study population

**Fig. 1:**
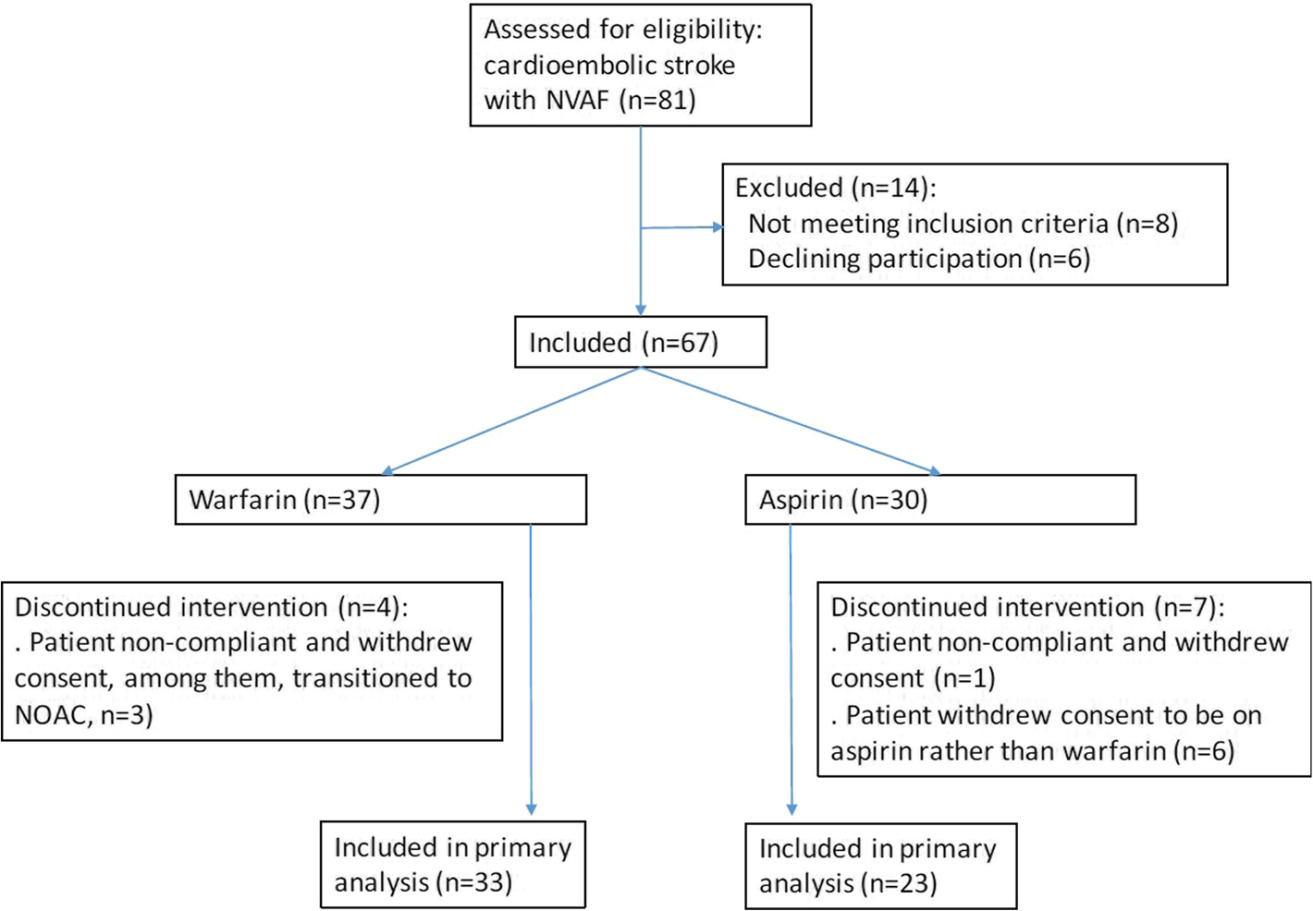
Flowchart of patient’s selection

Total 67 patients were included, and 56 completed the trial (33 warfarin and 23 aspirin). Eleven patients were dropped out after study inclusion due to noncompliance, withdrawal of consent, and transition to other drugs (Fig. 1). When comparing dropouts and patients completing the study, there was no significant differences in age, sex, CHA_2_DS_2_-VASc scores, or treatment arm assignment (data are not shown, all *p* values> 0.1).

### Basic characteristics

There was no significant difference between the warfarin and aspirin group, including demographic and laboratory findings, as shown in Table 1. As for clinical profiles, all the NIHSS, mRS, CHADS_2_ or CHA_2_DS_2_-VASc scores did not show any significant difference between the two groups.

**Table 1:** Demographics of patients with ischemic stroke patients

Variables^a^	Aspirin versus warfarin		*p* value*
	Aspirin (*n* = 23)	Warfarin (*n* = 33)	
Age, years	72.2 ± 8.6	74.2 ± 8.3	0.389
Female, *n* (%)	11 (47.8)	17 (51.5)	0.786
Clinical Profiles			
CHADS_2_	3.2 ± 0.9	3.5 ± 0.8	0.232
CHA_2_DS_2_-VASc	4.4 ± 1.3	4.9 ± 1.2	0.238
NIHSS, initial	6.4 ± 6.2	5.6 ± 6.0	0.613
mRS, initial	2.3 ± 1.7	2.5 ± 1.7	0.669
Laboratory Data			
White blood cell, × 10^3^/μl	7.8 ± 2.7	7.5 ± 3.2	0.716
Hematocrit, %	37.0 ± 6.3	37.4 ± 5.7	0.803
Platelet, ×10^3^/μl	192.8 ± 44.1	219.9 ± 59.1	0.068
PT, %	93.1 ± 12.0	85.0 ± 12.5	0.051
aPTT, second	27.1 ± 7.6	30.7 ± 18.1	0.371
D-dimer, mg/L	2.4 ± 3.6	2.4 ± 2.7	0.959
Albumin, g/dL	5.6 ± 8.8	5.2 ± 7.2	0.854
Total protein, g/dL	6.6 ± 0.8	6.7 ± 0.8	0.479
Creatinine, mg/dL	0.8 ± 0.3	1.3 ± 2.4	0.296
eGFR, mL/min/1.73m^2^	84.7 ± 19.0	85.1 ± 17.8	0.940
Total cholesterol, mg/dL	153.1 ± 30.2	155.3 ± 28.0	0.776
Triglyceride, mg/dL	95.2 ± 39.8	90.4 ± 36.3	0.638
High density lipoprotein, mg/dL	47.2 ± 13.9	47.5 ± 14.2	0.951
HbA1c, %	5.9 ± 0.4	6.0 ± 0.5	0.763
UACR, mg/g	114.8 ± 116.8	160.8 ± 203.8	0.334
Homocysteine, μmol/L	16.2 ± 4.6	17.5 ± 13.1	0.654

*Independent T-test was used for numerical values and chi square test for categorical value

^a^ Values are presented mean ± SD, unless indicated otherwise

CHADS_2_: *C* congestive heart failure, *H* hypertension, *A* ≥75 years; *D* diabetes mellitus, *S*_*2*_ prior stroke, transient ischemic attack or thromboembolism, CHA_2_DS_2_-VASc: *C* congestive heart failure, *H* hypertension, *A*_*2*_ ≥75 years, *D* diabetes mellitus, *S*_*2*_ prior stroke, transient ischemic attack or thromboembolism, *V* vascular disease, *A* 65–74 years, *Sc* sex category, *NIHSS* National Institutes of Health Stroke Scale, *mRS* modified Rankin Scale, *PT* prothrombin time, *aPTT* activated partial thromboplastin time, *BUN* blood urea nitrogen, *eGFR* estimated glomerular filtration rate, *UACR* urine albumin-to-creatinine ratio

### Blood viscosity of warfarin and aspirin

**Fig. 2:**
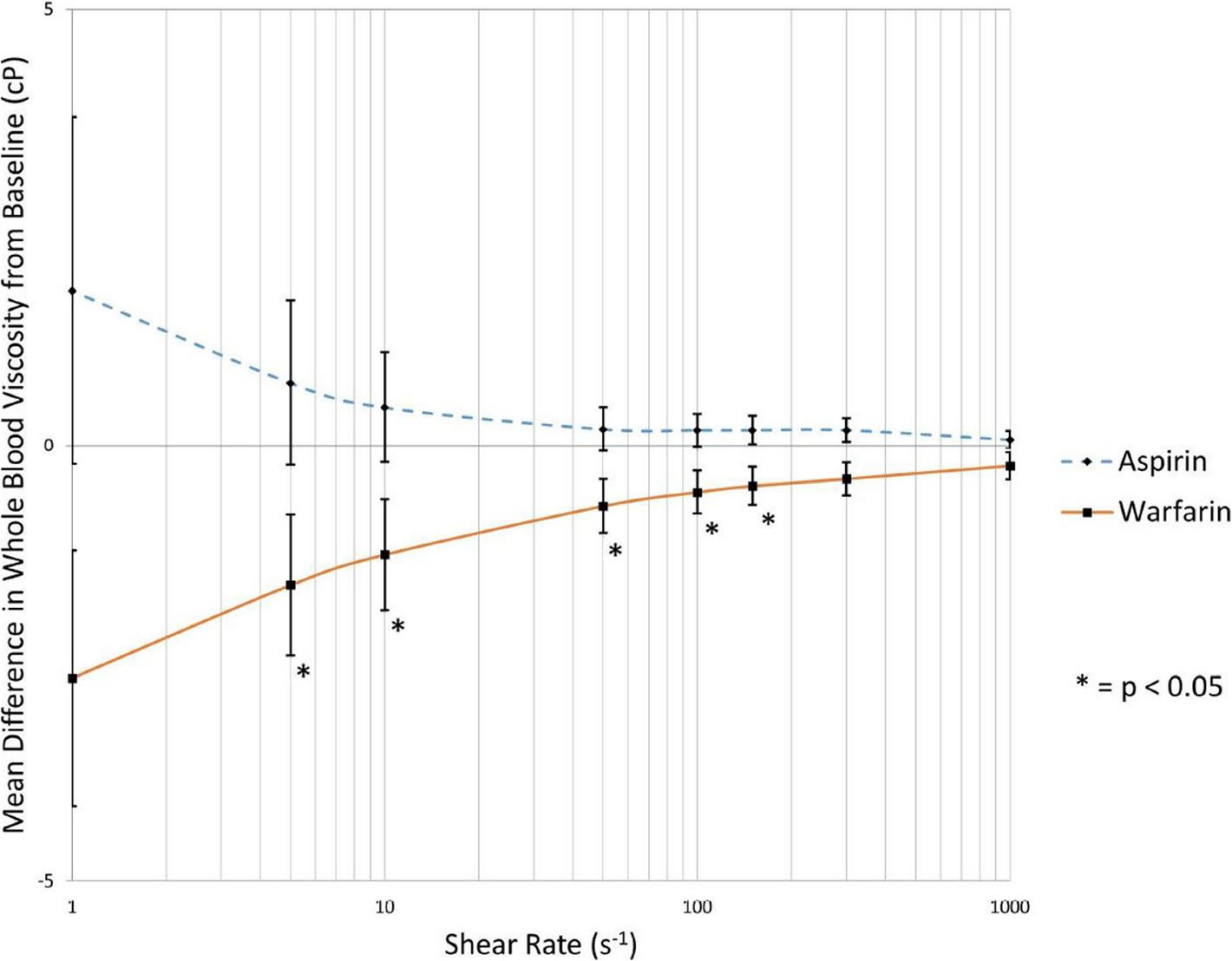
Mean difference of warfarin and aspirin in whole blood viscosity from baseline. Warfarin numerically reduced post-treatment blood viscosity at all shear rates

There was no significant difference of baseline levels of WBV between the two groups (Table 2). In the warfarin group, the INR was 1.12 ± 0.19 (mean ± SD) versus 2.37 ± 0.48 (*p* < 0.001) before and after warfarin administration. After the treatments, warfarin numerically reduced post-treatment blood viscosity at all shear rates when INR reached target ranges (Fig. 2, Table 3). These differences were statistically significant at shear rates of 150 (*p* = 0.032), 100 (*p* = 0.022), 50 (*p* = 0.014), 10 (*p* = 0.033), and 5/s (*p* = 0.034). At shear rates of 300 and 1/s, marginal significances were observed (*p* = 0.055 and 0.071, respectively). In contrast, aspirin had no effect on blood viscosity compared to pre-treatment state (*p* > 0.05, Table 4).

**Table 2:** Baseline whole blood viscosity of aspirin and warfarin group

Shear rate	Whole blood viscosity (cP, baseline)		*p* value^§^
	Aspirin group	Warfarin group	
1000/s	3.39	3.57	0.835
300/s	3.81	3.81	0.910
150/s	4.17	4.17	0.977
100/s	4.41	4.31	0.953
50/s	4.97	4.84	0.871
10/s	7.43	7.67	0.865
5/s	9.7	10.42	0.768
1/s	23.01	25.53	0.535

^§^Independent T-test was used

**Table 3:** The difference in blood viscosity before and after treatment in the warfarin group

Shear rate	Whole blood viscosity (cP, baseline)		*p* value^§^
	Baseline (mean ± SD)	After treatment (mean ± SD)	
1000/s	3.88 ± 0.99	3.64 ± 0.60	0.135
300/s	4.29 ± 1.21	3.90 ± 0.63	0.047
150/s	4.61 ± 1.42	4.14 ± 0.70	0.039
100/s	4.85 ± 1.60	4.31 ± 0.76	0.036
50/s	5.44 ± 2.02	4.74 ± 0.93	0.031
10/s	8.77 ± 4.06	7.52 ± 1.75	0.058
5/s	11.67 ± 5.21	10.05 ± 2.54	0.056
1/s	27.41 ± 9.59	24.73 ± 7.79	0.078

^§^Paired T-test was used. *SD* standard deviation

**Table 4:** The difference in blood viscosity before and after treatment in the aspirin group

Shear rate	Whole blood viscosity (cP, baseline)		*p* value^§^
	Baseline (mean ± SD)	After treatment (mean ± SD)	
1000/s	3.51 ± 0.62	3.58 ± 0.52	0.498
300/s	3.81 ± 0.75	3.99 ± 0.61	0.199
150/s	4.09 ± 0.87	4.27 ± 0.72	0.273
100/s	4.31 ± 0.98	4.49 ± 0.83	0.344
50/s	4.85 ± 1.26	5.04 ± 1.09	0.467
10/s	7.72 ± 2.57	8.16 ± 2.55	0.495
5/s	10.22 ± 3.62	10.94 ± 3.85	0.454
1/s	23.95 ± 8.90	25.73 ± 8.46	0.383

^§^Paired T-test was used

Blood viscosity decreased by more than 1 cP at each shear rate was also evaluated using a multiple logistic regression model. At shear rates of 300, 150, 5, and 1/s, the blood viscosity was independently and significantly associated with warfarin compared to aspirin after adjusting for age, sex, CHA_2_DS_2_-VASc scores, and baseline hematocrit (Table 5).

**Table 5:** Multivariate^a^ association between blood viscosity decrease more than 1 cP and warfarin (compared to aspirin)

Shear rate	Blood viscosity decrease more than 1 cP		*p* value
	Odds ratio	95% CIs	
1000/s	0.28	0.08–1.01	0.051
300/s	3.71	1.04–13.17	0.043
150/s	4.30	1.20–15.49	0.026
100/s	3.37	0.99–11.50	0.053
50/s	2.72	0.86–8.60	0.089
10/s	2.81	0.84–9.39	0.093
5/s	3.35	1.00–11.25	0.050
1/s	4.38	1.31–14.64	0.017

^a^Adjusted for age, sex, CHA_2_DS_2_-VASc, and hematocrit

## Discussion

This study investigated the effect of warfarin or aspirin on the WBV in cardioembolic stroke patients with NVAF. There was no difference in blood viscosity after the administration of aspirin, but the viscosity of the warfarin group decreased at all shear rates. This is the first study to reveal the relationship between the WBV and warfarin in patients with atrial fibrillation. The WBV has been thought of as an emerging risk marker for vascular diseases and mortality [19, 20]. Therefore, anti-viscogenic effects of warfarin on WBV may be considered carefully.

The associations between cardiovascular risk factors and the WBV were reported previously [21]. As for stroke, there have been several reports on the relationship between stroke and blood viscosity [22, 23]. Relative blood viscosity (WBV divided by plasma viscosity) in the patients with ischemic stroke was increased in the acute phase [24]. The British Regional Heart Study, with 9.5 years of follow up, showed that the high hematocrit level increased the incidence of stroke [25]. The severity of spontaneous echo contrast (SEC) in patients with acute or chronic cerebrovascular disease was associated with increased plasma and serum viscosity [26].

Anticoagulants, such as warfarin or heparin, inhibit the activities of coagulation factors and prevent blood clotting. It has been reported for the relationship between the blood viscosity and some kinds of anticoagulants. Heparin and argatroban have been shown to decrease blood viscosity in a dose-dependent manner in healthy human samples [27, 28]. As for mucopolysaccharide polysulfate, the WBV was also decreased in healthy adults dose-dependently [29]. Although the present study was not designed to reveal the dose-dependent manner of WBV reduction by warfarin, the WBV has been reported to be reduced with anticoagulants. The relationship between non-vitamin K dependent anticoagulant and the WBV in atrial fibrillation has not reported yet.

Compared to anticoagulants, antiplatelet agents showed various responses. As for aspirin, the previous study showed that the WBV did not change at any dose of aspirin [13]. In the present study, the blood viscosity values at all measured shear rates did not show any significant change after aspirin treatment, which was similar with previous studies. Other prior reports have shown the effects of various antiplatelet agents on WBV; no response with aspirin and cilostazol [30], but lowering responses with dipyridamole and clopidogrel on WBV [18, 31]. Clinical trials of antiplatelet agents or their combinations have been performed for their effectiveness on ischemic cardiocerebrovascular diseases or for safety issues. The antiplatelet agents or their combinations showed diverse clinical outcome according to cardio- or cerebrovascular diseases [32, 33]. Given that the WBV responses, it might be considered whether WBV can be adjusted according to target organ or disease categories.

Warfarin showed superiority to aspirin in the patients with ischemic stroke and valvular or non-valvular AF [34]. Considering the WBV-lowering effect of warfarin, this suggests that the WBV effect may be more important in cardiogenic stroke including AF than non-cardiogenic stroke, such as large artery atherosclerosis. The pathophysiological mechanism that causes the ischemic stroke in cardiogenic stroke is multifactorial but can be described conceptually in relation to Virchow’s triad for thrombogenesis. These components comprise abnormal blood stasis in the atria, structural heart disease and abnormalities of blood coagulation [35]. Atrial fibrillation causes a structural change in left atrium (LA) and appendage, leading to a larger diameter, which increases the likelihood of blood stasis. A previous study reported that the size of the LA corrected by body surface area is an independent risk factor for stroke occurrence [36]. Atrial fibrillation also changes the composition of blood clotting factors, including fibrin and thrombin-antithrombin complexes, which promote thrombus formation [37]. These hemorrheological changes in AF might be improved by warfarin with reduced effects of WBV.

The WBV provides an absolute value, rather than a relative comparison to a reference like INR. It implies that the WBV could be used to stratify and predict the risk of the future ischemic cerebrovascular diseases. Previous reports on normative data might be useful to identify the abnormal distributions of the WBV in patients with vascular diseases [38]. When analyzing the WBV, the principal determinants which include hematocrit, red blood cell deformability, and plasma viscosity might be considered as well.

Limitations of the present study includes: first, the relatively small sample size with modest drop rates. However, compared to the previous studies in which healthy individuals were enrolled, the present study was performed in the patients with acute ischemic stroke. The higher drop rate in the aspirin arm, which could increase the chance of type 2 error, might be caused by the selection only in the patients. Second, the WBV was measured when INR reached target ranges in the warfarin group, so we could not show whether the WBV could be reduced further at higher concentrations of warfarin. Lastly, since this study was conducted in Asia, that is, in Korea, it is premature to generalize the effect of warfarin on the WBV on global populations. To clarify the relationship between warfarin and the WBV, subsequent multi-ethnic studies are needed.

In conclusion, warfarin reduced the WBV significantly in patients with acute ischemic stroke with NVAF, compared to aspirin. The reduction in blood viscosity with warfarin may provide an integrated measure on thrombotic risk. Further studies are needed to evaluate the usefulness of WBV in the prediction of stroke at the time of clinical presentation, and the effects of WBV reductions with warfarin therapy on the risk of recurrent stroke in various etiologies of ischemic stroke including cardiogenic stroke.

## Abbreviations

AF: atrial fibrillation
BV: blood viscosity
INR: international normalized ratio
LA: left atrium
mRS: modified Rankin Scale
NIHSS: National Institute of Health Stroke Scale
NOAC: non–vitamin K antagonist oral anticoagulant
NVAF: non-valvular atrial fibrillation
SLE: Systemic Lupus Erythematosus
